# Adipogenic Differentiation of hMSCs is Mediated by Recruitment of IGF‐1r Onto the Primary Cilium Associated With Cilia Elongation

**DOI:** 10.1002/stem.1975

**Published:** 2015-05-21

**Authors:** Melis T. Dalbay, Stephen D. Thorpe, John T. Connelly, J. Paul Chapple, Martin M. Knight

**Affiliations:** ^1^ Institute of Bioengineering and School of Engineering and Materials Science, Queen Mary University of LondonLondonUnited Kingdom; ^2^ Institute of Bioengineering and Institute of Cellular and Molecular Sciences, Blizard Institute, Barts and The London School of Medicine and Dentistry, Queen Mary, University of LondonLondon, UK; ^3^ Centre for EndocrinologyWilliam Harvey Research Institute, Barts and the London School of Medicine and Dentistry, Queen Mary, University of LondonLondonUK

**Keywords:** Human mesenchymal stem cell, Cilia, Adipogenic differentiation, Chondrogenic differentiation, Osteogenic differentiation, IGF‐1 receptor

## Abstract

Primary cilia are single non‐motile organelles that provide a highly regulated compartment into which specific proteins are trafficked as a critical part of various signaling pathways. The absence of primary cilia has been shown to prevent differentiation of human mesenchymal stem cells (hMSCs). Changes in primary cilia length are crucial for regulating signaling events; however it is not known how alterations in cilia structure relate to differentiation. This study tested the hypothesis that changes in primary cilia structure are required for stem cell differentiation. hMSCs expressed primary cilia that were labeled with acetylated alpha tubulin and visualized by confocal microscopy. Chemically induced differentiation resulted in lineage specific changes in cilia length and prevalence which were independent of cell cycle. In particular, adipogenic differentiation resulted in cilia elongation associated with the presence of dexamethasone, while insulin had an inhibitory effect on cilia length. Over a 7‐day time course, adipogenic differentiation media resulted in cilia elongation within 2 days followed by increased nuclear PPARγ levels; an early marker of adipogenesis. Cilia elongation was associated with increased trafficking of insulin‐like growth factor‐1 receptor β (IGF‐1Rβ) into the cilium. This was reversed on inhibition of elongation by IFT‐88 siRNA transfection, which also decreased nuclear PPARγ. This is the first study to show that adipogenic differentiation requires primary cilia elongation associated with the recruitment of IGF‐1Rβ onto the cilium. This study may lead to the development of cilia‐targeted therapies for controlling adipogenic differentiation and associated conditions such as obesity. Stem Cells *2015;33:1952–1961*

## Introduction

Primary cilia are single non‐motile organelles that typically protrude from the cell surface and are expressed by most mammalian cell types including stem cells 1. They play a critical role in the regulation of cell and tissue development and homeostasis 2, 3 via a variety of signaling pathways including Wnt 4, hedgehog 5, 6, notch 7, mechanotransduction 8, and growth factor signaling 9.

Primary cilia consist of a microtubule structure called the axoneme that is covered by a specialized lipid bilayer. The axoneme extends from the basal body, which originates from the most mature of the two centrioles. Proteins are transported on and off the cilium by molecular motors through the process of intraflagellar transport (IFT) 10. Cilia assembly and disassembly is, therefore, regulated by IFT, which controls the transport of tubulin to and from the tip of the cilium and hence controls the cilia length 11. Transport of structural proteins, IFT is also involved in trafficking of cilia signaling proteins. This process is critical to the role of primary cilia in acting as a signaling hub that modulates mechanical, osmotic, and chemical signaling 12. Mutations occurring in IFT proteins may, therefore, influence cilia structure and impair signaling pathways, resulting in a range of developmental and degenerative disorders, collectively termed ciliopathies 13.

Recent studies have shown that differentiation of mesenchymal stem cells requires the presence of primary cilia and associated IFT. In particular, primary cilia are involved in differentiation along osteogenic 8, 14, 15, 16, 17, 18, adipogenic 16, 17, 19, chondrogenic 17, 20, and neurogenic 21 lineages. Interestingly, the length of primary cilia differs between various differentiated cell types. For example, epithelial cells typically have longer cilia than articular chondrocytes. However, it is not known how primary cilia structure changes during differentiation and whether any such changes are important in the regulation of differentiation. Recent evidence suggests that the length of primary cilia influences cilia signaling pathways including mechanotransduction, hedgehog, and wnt signaling 22, 23, 24. The mechanisms through which this occurs are unclear. Besschetnova *et al*. have demonstrated that anterograde IFT velocity is increased in longer cilia 25; however Ludington *et al*. show that this is associated with a reduction in the injection of IFT cargoes thereby affecting the trafficking of signaling molecules and receptors into the ciliary compartment 26. In addition, other studies show that the process of cilia elongation or shortening also regulates cilia function associated with dynamic changes in trafficking 27.

Therefore, we hypothesize that biochemical differentiation conditions induce alterations in primary cilia length and these structural changes are important in facilitating the differentiation of human bone marrow‐derived mesenchymal stem cells (hMSCs). We show that the differentiation of hMSCs results in lineage specific changes in cilia length and prevalence. We then focus on adipogenic differentiation conditions that increase cilia length and demonstrate that elongation and associated alterations in trafficking are required for adipogenesis. Finally, we examine the mechanism involved and reveal that cilia elongation drives increased ciliary expression of IGF‐1R and that subtle inhibition of this elongation by knock down of IFT88 prevents adipogenic differentiation.

## **Materials and Methods**

### hMSC Culture and Differentiation

hMSCs were obtained from Stem Cell Technologies (Manchester, UK, www.stemcell.com) and maintained in basal media (BM) consisting of low glucose Dulbecco's modified Eagle Medium (DMEM; Gibco, Paisley, UK, www.lifetechnologies.com) with 10% fetal bovine serum (FBS) and 1% penicillin–streptomycin (both Sigma‐Aldrich, Dorset, UK, www.sigmaaldrich.com), with the addition of 1 ng/ml fibroblast growth factor‐2 (FGF‐2; PeproTech, London, UK, www.peprotech.com). Media was exchanged every 3–4 days. For all studies, hMSCs at passage 4–7 were plated at 5 × 10^3 ^cells per square centimeter in either 24 well plates on FBS‐coated glass coverslips (thickness 1.5) for imaging studies or in 6‐well plates for protein isolation. Five days post seeding, media was changed to differentiation media. Adipogenic differentiation media (AM) consisted of BM with the addition of 1 μM dexamethasone, 500 μM 3‐Isobutyl‐1‐methylxanthine (IBMX), 100 μM indomethacin, and 10 μg/ml insulin (all Sigma‐Aldrich, Dorset, UK, www.sigmaaldrich.com). Osteogenic media (OM) consisted of BM with the addition of 100 nM dexamethasone, 50 μM l‐ascorbic acid, and 10 mM β‐glycerophosphate (all Sigma‐Aldrich, Dorset, UK, www.sigmaaldrich.com). Chondrogenic media (CM+) consisted of high glucose DMEM (Gibco) with 1% penicillin–streptomycin, 1 mM sodium pyruvate, 1.5 mg/ml bovine serum albumin (BSA), 40 μg/ml l‐proline, 4.7 μg/ml linoleic acid, 50 μg/ml l‐ascorbic acid, 100 nM dexamethasone (all Sigma‐Aldrich, Dorset, UK, www.sigmaaldrich.com), and 1X insulin‐transferrin‐selenium G supplement (Gibco, Paisley, UK, www.lifetechnologies.com) with the addition of 10 ng/ml transforming growth factor‐β3 (TGF‐β3; PromoKine, Heidelberg, Germany, www.promokine.info). CM without TGF‐β3 (CM‐) was used as a control for chondrogenic differentiation. Media was exchanged every 3–4 days.

### siRNA Transfection

Transfections were performed on hMSCs 5 days post seeding in antibiotic free BM using DharmaFECT 1 transfection reagent (GE Dharmacon, Lafayette, CO, USA, dharmacon.gelifesciences.com) according to manufacturer's protocol. hMSCs were transfected with 25 nM SMART pool ON‐TARGETplus human siRNA to intraflagellar transport protein‐88 (*IFT88*) or ON‐TARGETplus non‐targeting pool siRNA (both GE Dharmacon, Lafayette, CO, USA, dharmacon.gelifesciences.com). Transfection media was exchanged for AM 24 hours post transfection. At days 2 and 5 of adipogenic differentiation induction, hMSCs were either fixed for immunocytochemistry or lysed for protein analysis. For day 5 samples, transfections were repeated in antibiotic‐free AM in the same manner 72 hours post the first transfection (day 2 of differentiation induction).

### Immunocytochemistry and Staining

To improve cilia prevalence in BM, OM, and AM cultures containing FBS 28, serum was withdrawn from culture media 24 hours before fixation as adopted in numerous previous studies. Cells were fixed in 4% PFA for 10 minutes and washed in phosphate‐buffered saline (PBS). To assess adipogenic differentiation, lipid droplets were stained using 0.2% oil red O in 60% isopropanol for 5 minutes. Osteogenic differentiation was assessed by detection of alkaline phosphatase activity. In this case, coverslips were fixed using 4% paraformaldehyde (PFA) for 2 minutes, washed in PBS, and immediately stained using an alkaline phosphatase detection kit according to manufacturer's instructions (Merck Millipore, Watford, UK, www.merckmillipore.com). Chondrogenic differentiation was assessed via collagen type II immunohistochemistry. For this and other immunohistochemistry staining, fixed cells were washed with PBS and permeabilized with 0.5% Triton‐X100 for 10 minutes and washed with PBS. Samples were blocked in 5% goat serum with 1 mg/ml BSA in PBS for 30 minutes at room temperature followed by incubation with primary antibodies at 4°C overnight. Primary antibodies used were mouse monoclonal anti acetylated α‐tubulin (1:2000; Sigma‐Aldrich, Dorset, UK, www.sigmaaldrich.com), rabbit polyclonal anti‐pericentrin (1:500; Abcam, Cambridge, UK, www.abcam.com), rabbit polyclonal anti‐collagen type II (1:100; Abcam, Cambridge, UK, www.abcam.com), mouse monoclonal anti‐Ki67 (1:500; Sigma‐Aldrich, Dorset, UK, www.sigmaaldrich.com), rabbit polyclonal anti‐peroxisome proliferator‐activated receptor γ (PPARγ; 1:100; Santa Cruz Biotechnology, Heidelberg, Germany, www.scbt.com), and rabbit polyclonal anti‐IGF‐1Rβ (1:100; Santa Cruz Biotechnology, Heidelberg, Germany, www.scbt.com). Secondary antibodies were Alexa Fluor 488 F(ab')_2_ fragment of goat anti‐mouse IgG (H+L), Alexa Fluor 488 goat anti‐rabbit IgG (H+L), Alexa Fluor 594 F(ab')_2_ fragment of goat anti‐rabbit IgG (H+L), and Alexa Fluor 633 goat anti‐rabbit IgG (H+L) (all 1:1000; Molecular Probes, Paisley, UK, www.lifetechnologies.com). F‐actin was stained using Alexa Fluor 555 phalloidin (1:50), and nuclei were stained with Hoechst 33342 (1:5000; both Molecular Probes, Paisley, UK, www.lifetechnologies.com). Immunofluorescently stained coverslips were mounted using ProLong Gold Antifade Mountant (Molecular Probes, Paisley, UK, www.lifetechnologies.com).

### Confocal Microscopy

Stained coverslips were imaged using a Leica SP2 confocal microscope (Leica Microsystems, Milton Keynes, UK, www.leica‐microsystems.com), with multiple z‐sections taken through the thickness of the cell at 500 nm intervals. Maximum projections of z‐stacks were imported into image J for measurement of cilia length using acetylated α‐tubulin and pericentrin to identify primary cilia. Cilia length is given in micrometers (μm). Image J was also used to quantify ciliary IGF‐1Rβ and nuclear PPARγ intensity by manually tracing around each cilium and nucleus and measuring the mean intensity in the corresponding fluorescent channel. Intensity values are presented in arbitrary units (AU) as obtained from the confocal software. Mean values of 100–110 measurements per sample were plotted in each graph.

### Western Blots

hMSCs were lysed in radio‐immunoprecipitation assay (RIPA) buffer (New England Biolabs, Herts, UK, www.neb.com) containing protease inhibitors (Roche Diagnostics, Burgess Hill, UK, www.roche.co.uk). Total protein was measured using Bradford reagent (Sigma‐Aldrich, Dorset, UK, www.sigmaaldrich.com). Protein supernatants were reduced in Lamelli buffer, separated on a Mini‐PROTEAN TGX Any kD gel and transferred to nitrocellulose membranes (all Bio‐Rad Laboratories, Hemel Hempstead, UK, www.bio‐rad.com). Membranes were blocked in 5% milk for 60 minutes before overnight incubation with primary antibodies; rabbit polyclonal anti‐PPARγ (1:500; Santa Cruz Biotechnology, Heidelberg, Germany, www.scbt.com), rabbit polyclonal anti‐IFT88 (1:500; Proteintech, Manchester, UK, www.ptglab.com), and mouse monoclonal anti‐β actin (1:10,000 Abcam, Cambridge, UK, www.abcam.com). Secondary antibodies used were IRDye 680RD goat anti‐mouse IgG (H+L) and IRDye 800CW donkey anti rabbit IgG (H+L) (both 1:15,000; LI‐COR Biotechnology, Cambridge, UK, www.licor.com/bio). Membranes were imaged using an Odyssey infra‐red imaging system (LI‐COR Biotechnology, www.licor.com/bio).

### Statistics

Statistical analyses were performed using GraphPad Prism (GraphPad Software, La Jolla, CA, USA, www.graphpad.com). When data sets adhered to a normal distribution, two‐sample *t*‐test or analysis of variance was used as indicated in figure legends. For nonparametric data sets, Mann–Whitney *U* tests were used to compare conditions. Data are presented as mean ± SE.

## Results

### Primary Cilia Length Changes During hMSC Differentiation in a Lineage Specific Manner

To investigate changes in primary cilia length and prevalence during differentiation, human bone marrow‐derived MSCs were seeded on glass coverslips and cultured in BM for 5 days. The media was then replaced with either AM, OM, CM‐, or CM+, with some samples maintained in BM as an undifferentiated control. As biochemical induction of chondrogenesis involves the use of a serum‐free media formulation containing TGF‐β3 (CM+), a TGF‐β3 free equivalent media (CM−) was used as a control for chondrogenic differentiation. Cells were cultured in differentiation conditions for a further 7 days before fixation (day 7). In addition, separate cells were fixed before differentiation (day 0). Differentiation along each lineage was confirmed at day 7 with adipogenic differentiation inducing lipid droplet accumulation demonstrated by oil red O staining, osteogenic hMSCs stained positive for alkaline phosphatase, and chondrogenic hMSCs stained positively for collagen type II (Supporting Information Fig. S1).

**Figure 1:**
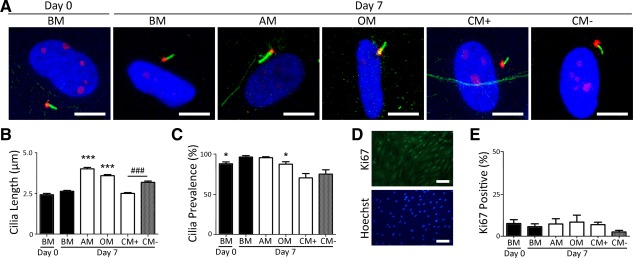
Primary cilia length changes during human mesenchymal stem cell (hMSC) differentiation in a lineage specific manner. **(A):** Representative maximum projection confocal images of hMSC primary cilium at days 0 and 7 of culture in basal media (BM), adipogenic media (AM), osteogenic media (OM), chondrogenic media (CM) without TGF‐β3 (CM‐), and CM with TGF‐β3 (CM+). Primary cilia were labeled for acetylated α‐tubulin (green), the basal body stained for pericentrin (red), while nuclei were stained with Hoechst 33342 (blue). Scale bar = 10 µm. **(B):** Primary cilia length at days 0 and 7 of differentiation induction. *n* = 100 cilia measured per group. ***, *p* < 0.001 versus day 7 BM; ###, *p* < 0.001 versus day 7 CM‐; Mann–Whitney *U* test. **(C):** Corresponding cilia prevalence at days 0 and 7 of differentiation induction. *n* = 5 fields per condition; *, *p* < 0.05 versus day 7 BM; Mann–Whitney *U* test. **(D):** Representative immunofluorescent staining of Ki67 (green, top) and DNA (blue, bottom). Scale bar = 100 μm. **(E):** Quantification of cell proliferation by Ki67 nuclear staining in hMSCs at day 7 of differentiation. *n* = 5 fields per condition with >100 cells per field. A one‐way analysis of variance revealed no significant differences in proliferation rate between the conditions. Abbreviations: AM, adipogenic media; BM, basal media; CM, chondrogenic media; OM, osteogenic media.

Primary cilia were labeled with acetylated α‐tubulin (green) and the basal body with pericentrin (red) (Fig. 1A). While there was no significant change in cilia length for hMSCs cultured in BM over the 7‐day period, primary cilium length was observed to increase by day 7 following differentiation in OM and AM. Chondrogenic differentiation media (CM+) produced no change in length compared with basal control. However, when compared with the TGF‐β3‐free chondrogenic control media (CM−), chondrogenic differentiation in the presence of TGF‐β3 significantly shortened the primary cilium (*p* < 0.001; Fig. 1B). At day 0, 87.8 ± 2.4% of hMSCs expressed a primary cilium and this increased to 96.4 ± 1.6% over the 7‐day culture period (*p* < 0.05; Fig. 1C). At day 7, adipogenic differentiation resulted in no change in percentage ciliation. By contrast, osteogenic differentiation resulted in decreased ciliation to a mean value of 87.4 ± 3.0%. Cells grown in CM with or without TGF‐β3 displayed the greatest reduction in primary cilia expression with 75 ± 5.4% and 70.4±5.3% of cells presenting a primary cilium, respectively (Fig. 1C). As cilia are resorbed at cell division 29, we investigated whether the observed differences in ciliation were associated with corresponding differences in hMSC proliferation. Based on Ki67 expression, hMSC proliferation was consistently below 10% with no significant differences in proliferation between any of the groups (Fig. 1E), indicating that changes in primary cilium length during differentiation were not the result of differences in hMSC proliferation.

### Nuclear Localization of PPARγ Increases Following Primary Cilia Elongation During Adipogenic Differentiation

**Figure 2:**
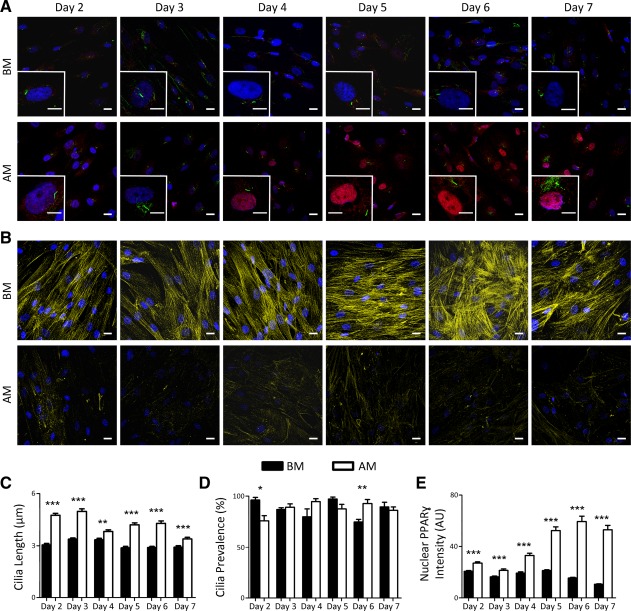
Time course showing nuclear peroxisome proliferator‐activated receptor γ (PPARγ) localization which increases following primary cilia elongation over a 7‐day culture period in adipogenic media. **(A):** Representative maximum projection confocal images of human mesenchymal stem cells (hMSCs) grown in basal media (BM; top row) and adipogenic media (AM; bottom row) with an enlarged single cell (inset) from days 2 to 7. PPARγ is shown in red, acetylated α‐tubulin in green, and Hoechst stained nuclei in blue. Scale bar = 20 µm for field views and 10 µm for inset images. **(B):** Representative confocal images showing F‐actin (yellow) in hMSCs cultured in BM (top row) and in AM (bottom row) from day 2 to day 7. Hoechst stained nuclei in blue. Scale bar = 20 µm. Primary cilia length **(C)**, prevalence **(D),** and nuclear PPARγ intensity **(E)** for hMSCs cultured in either BM or AM. *n* = 100–110 cilia in (C); *n* = 5 fields in (D); *n* = 100–110 nuclei in (E). *, *p* < 0.05 versus BM; **, *p* < 0.01 versus BM; ***, *p* < 0.001 versus BM; Mann–Whitney *U* test. Abbreviations: AM, adipogenic media; BM, basal media; PPARγ, peroxisome proliferator‐activated receptor γ.

To investigate the role of primary cilia length changes on hMSC differentiation, we focused on differentiation down the adipogenic lineage that was associated with approximately 60% cilia elongation compared with undifferentiated hMSCs. We began by investigating cilia length and associated changes in the nuclear expression of the adipogenic marker PPARγ with time over the first 7 days of differentiation (Fig. 2A). In BM, cilia length of hMSCs remained approximately constant with a mean length of approximately 3 μm. By contrast, after 2 days of adipogenic induction, primary cilia were significantly longer than for nondifferentiated hMSCs in BM and remained longer throughout the 7‐day culture period (Fig. 2A, 2C). However, there were time‐dependent fluctuations in cilia length in adipogenic conditions with reduced levels of cilia elongation at days 4 and 7 (Fig. 2C; Supporting Information Fig. S6A). While total PPARγ expression levels were similar in hMSCs grown in BM and AM (Supporting Information Fig. S2), we observed greater levels of nuclear PPARγ localization consistent with adipogenic differentiation at all time‐points (*p* < 0.001) with a marked increase from day 4 of differentiation following the initial increase in cilia length at days 2 and 3 (Fig. 2E). Thus, there is a temporal mismatch between cilia elongation and subsequent nuclear PPARγ expression, since elongation precedes subsequent differentiation. Indeed, there was no correlation between primary cilia length and nuclear PPARγ intensity on an individual cell basis at any single time point (Supporting Information Fig. S3).

Adipogenic differentiation was also associated with actin cytoskeletal changes. hMSCs grown in BM contained organized, dense F‐actin stress fiber bundles. By contrast, hMSCs cultured in AM did not exhibit such organized F‐actin bundles (Fig. 2B). These changes in actin organization associated with adipogenic differentiation are in agreement with previous reports 30, 31.

Cilia prevalence was maintained between 75 and 100% for both culture groups over the 7‐day period (Fig. 2D). Cilia prevalence was lowest at day 2 of adipogenic induction (75.8 ± 5.1%) and increased at later time points (Fig. 2D; Supporting Information S6B). Assessment of cell proliferation based on Ki67 expression showed that the rate of cell division remained less than 10% and was not significantly altered with culture duration or differentiation (Supporting Information Fig. S4). Thus, temporal changes in cilia length with adipogenic differentiation cannot be attributed to differences in cell proliferation.

### Role of AM Components in Cilia Elongation and Differentiation

**Figure 3:**
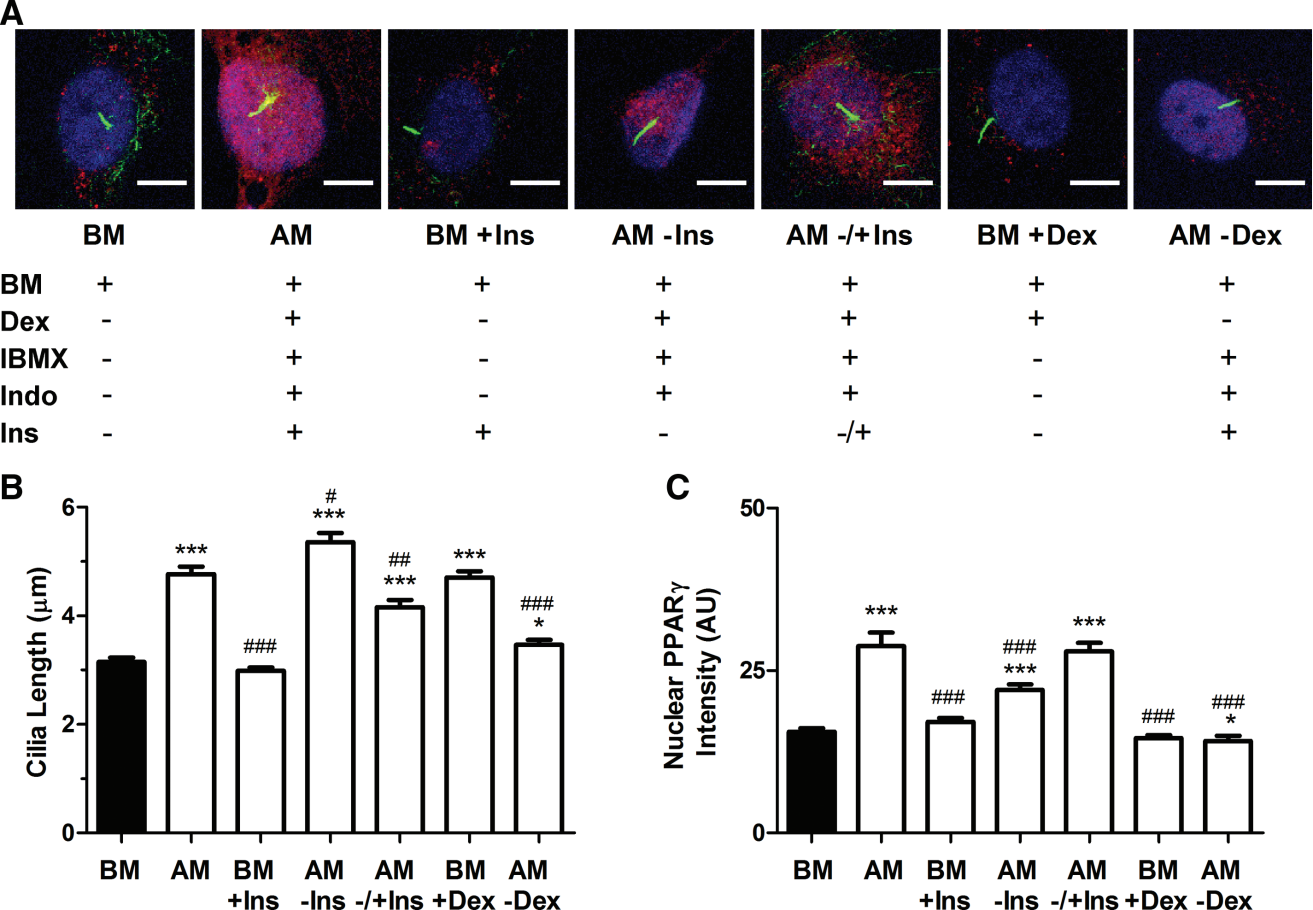
The role of media components in cilia elongation and adipogenic differentiation. **(A):** Representative confocal images showing primary cilia and nuclear peroxisome proliferator‐activated receptor γ (PPARγ) expression in human mesenchymal stem cells (hMSCs) cultured for 2 days in the presence of different media components: basal media (BM), complete adipogenic media (AM) including insulin, BM with insulin (BM+Ins), adipogenic media without insulin (AM − Ins), adipogenic media with no insulin for the initial 24 hours followed by addition of insulin for 24 hours (AM ± Ins), BM with dexamethasone alone (BM+Dex) and adipogenic media without dexamethasone (AM‐Dex). PPARγ is shown in red, acetylated α‐tubulin in green, and Hoechst stained nuclei in blue. Scale bar = 10 µm. Cilia length **(B)** and nuclear PPARγ intensity **(C)**. *n* = 100–110 cilia per group in (B) and *n* = 100–110 nuclei per group in (C). *, *p* < 0.05 versus BM; ***, *p* < 0.001 versus BM; #, *p* < 0.05 versus AM; ##, *p* < 0.01 versus AM; ### *p* < 0.001 versus AM; Mann–Whitney *U* test. Abbreviations: AM, adipogenic media; BM, basal media; Dex, dexamethasone; IBMX, 3‐Isobutyl‐1‐methylxanthine; Ins, insulin; PPARγ, peroxisome proliferator‐activated receptor γ.

To gain some insight into the mechanistic role of primary cilia elongation in adipogenic differentiation, we investigated the role of insulin on cilia length and prevalence. Insulin is a known stimulator of both insulin receptor (IR) and IGF‐1R signaling necessary for adipogenic differentiation. In particular, insulin is required for later events in adipogenesis such as maintenance of adipogenic phenotype by binding to IRs and IGF receptors (IGFR) 32, 33, 34. Here we cultured hMSCs for 48 hours in either BM or AM, both with (BM+Ins, AM) and without (BM, AM − Ins) insulin. An additional group (AM ± Ins) was cultured for 24 hours in insulin‐free AM (AM − Ins) followed by 24 hours in standard AM including insulin. In all cases, FBS was removed 24 hours before fixation. For hMSCs grown in AM, primary cilia length increased compared with cells in BM; however, primary cilia were significantly longer when insulin was not included in the AM (AM − Ins) (Fig. 3A, 3B). The addition of insulin after 24 hours (AM ± Ins) reduced cilia length compared with AM − Ins. Similarly, addition of insulin to BM alone (BM+Ins) did not induce any cilia elongation in contrast to the elongation observed in full AM (Fig. 3A, 3B). This increase in primary cilia length corresponded to increased nuclear PPARγ accumulation in cells that were grown in AM, AM − Ins, and AM ± Ins (Fig. 3A, 3C). In addition, hMSCs cultured in BM+Ins did not exhibit an increase in nuclear PPARγ associated with the absence of cilia elongation (Fig. 3A, 3C). Our data suggest that primary cilia elongation in the biochemical induction of adipogenic differentiation is due to one or a combination of dexamethasone, IBMX, and indomethacin, with insulin providing negative feedback on the regulation of primary cilia length.

The presence of dexamethasone in both AM and OM, both of which induced increased cilia length, prompted us to investigate whether dexamethasone alone would be able to induce primary cilia elongation. The presence of dexamethasone in BM (BM + Dex) was sufficient to induce primary cilia elongation to a similar level observed in full AM conditions (Fig. 3A, 3B). However, it was not sufficient to increase nuclear PPARγ levels (Fig. 3A, 3C). Similarly, culturing hMSCs in AM without dexamethasone substantially reduced the level of cilia elongation and did not increase nuclear PPARγ (Fig. 3A–3C). Cilia prevalence was similar across all groups (Supporting Information Fig. S5).

### Primary Cilia Elongation Increases IGF‐1Rβ Receptor Trafficking into the Cilium During Adipogenic Differentiation of hMSCs

**Figure 4:**
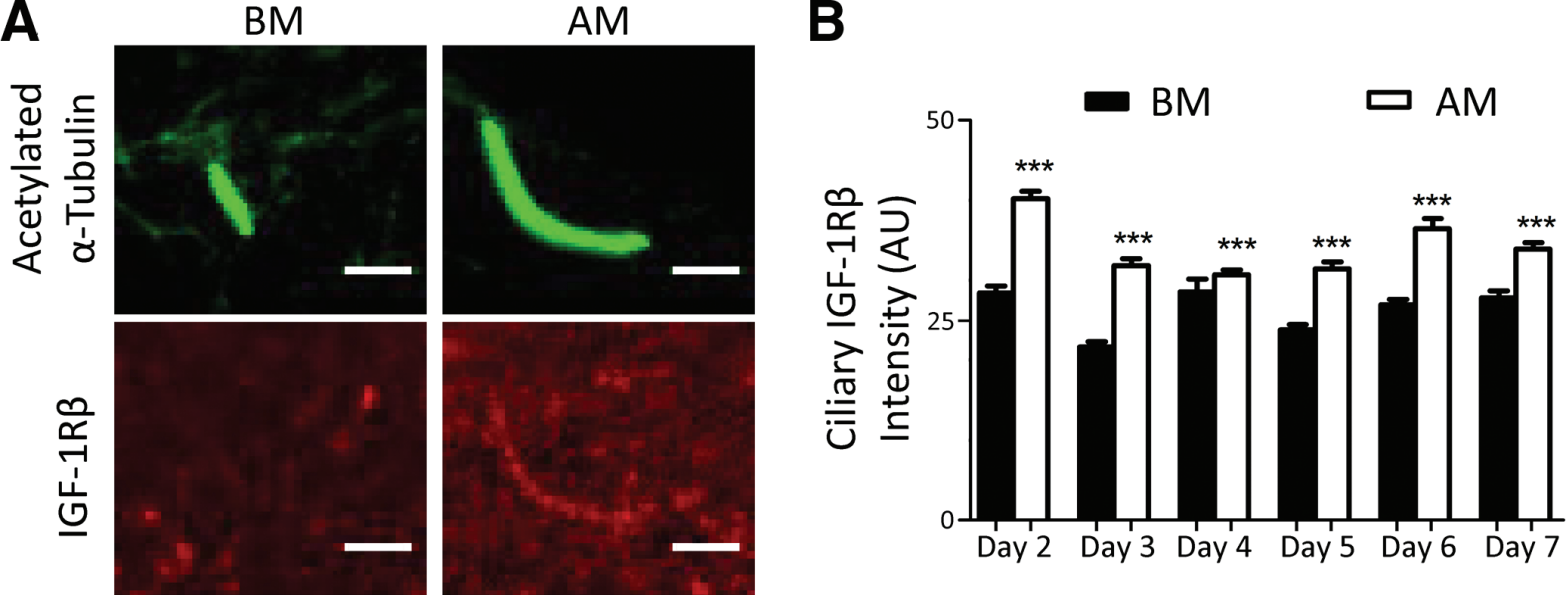
Cilia elongation in adipogenic media is associated with increased insulin‐like growth factor‐1 receptor β (IGF‐1Rβ) localization at the primary cilia. **(A):** Representative images of cilia labeled for acetylated α‐tubulin (top, green) on human mesenchymal stem cells (hMSCs) cultured in basal media (BM) and adipogenic media (AM) and corresponding images labeled for IGF‐1Rβ (bottom, red). Scale bar = 10 µm. **(B):** Ciliary IGF‐1Rβ intensity for BM and AM cultured hMSCs from days 2 to 7. *n* = 100–110 cilia measured per condition. ***, *p* < 0.001 versus BM; Mann–Whitney *U* test. Abbreviations: AM, adipogenic media; BM, basal media; IGF‐1Rβ, insulin‐like growth factor‐1 receptor β.

Zhu et al. have shown that initial activation of IGF‐1Rβ in response to insulin stimulation occurs on the primary cilium 32. Based on these findings and from our results showing the effects of AM components on cilia length, we hypothesized that primary cilia elongation may be associated with increased IGF‐1Rβ localization in the cilium and therefore may be necessary for adipogenic differentiation. To test this hypothesis, IGF‐1Rβ intensity in the primary cilia of hMSCs was assessed over a 7‐day period in BM and AM. Representative images of primary cilium (green) with corresponding images of IGF‐1Rβ localization (red) are presented in Figure 4A. IGF‐1Rβ intensity on the primary cilium (Fig. 4B) corresponds with the changes in primary cilia length during adipogenic differentiation (Supporting Information Fig. S6A), displaying the highest intensity at day 2 where cilia elongation was first observed. Ciliary IGF‐1Rβ intensity was greater in hMSCs grown in AM compared with cells grown in BM at all time‐points investigated. Although overall increases in cilia length corresponded to increases in ciliary associated IGF‐1Rβ, there was no correlation between primary cilia length and ciliary IGF‐R1β on an individual cell basis (data not shown). These results confirm that biochemical adipogenic induction drives cilia elongation and an associated recruitment of IGF‐1Rβ into the cilium of hMSCs.

### Primary Cilia Length Plays a Role in Regulating hMSC Adipogenic Differentiation Through Increased IGF‐1Rβ Trafficking into the Cilium

**Figure 5:**
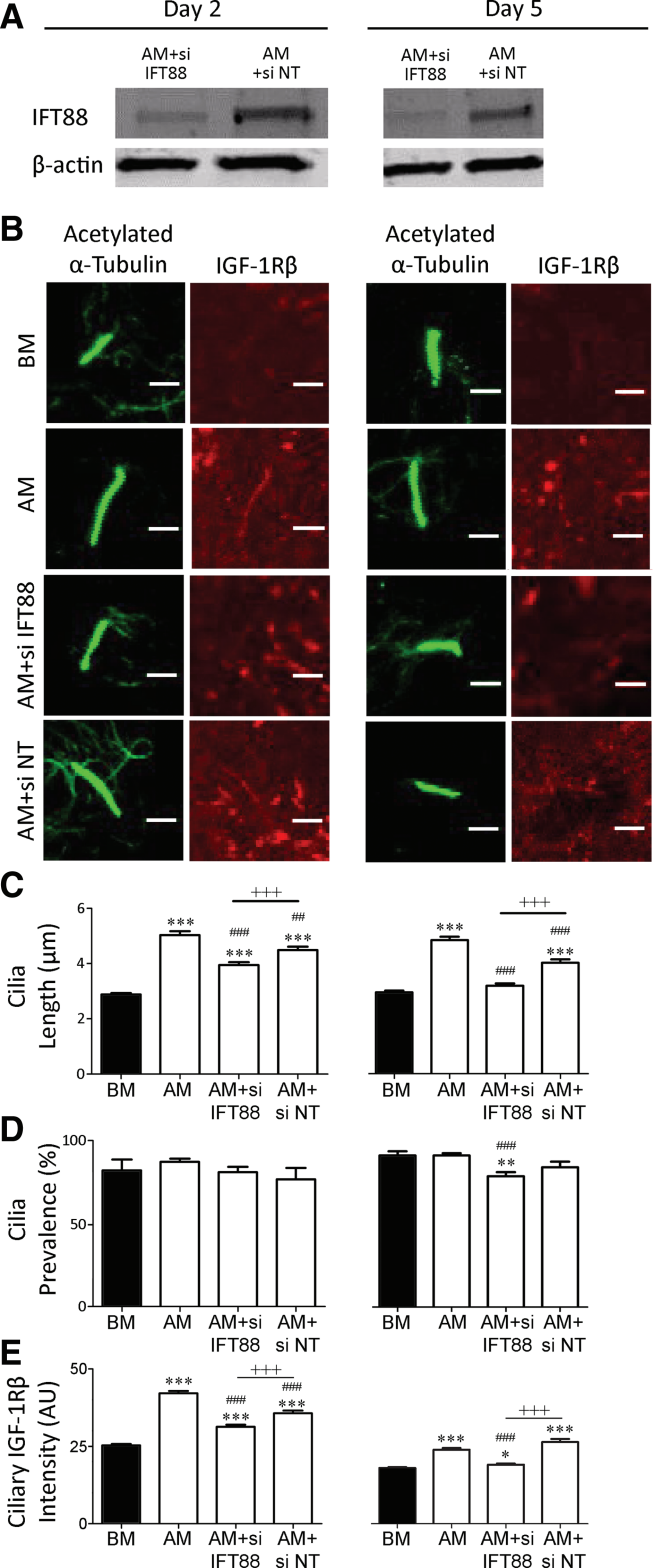
Intraflagellar transport protein‐88 (IFT88) siRNA prevents cilia elongation and reduces insulin‐like growth factor‐1 receptor β (IGF‐1Rβ) trafficking into the cilium. **(A):** Western blot for IFT88 and β‐actin protein for human mesenchymal stem cells (hMSCs) transfected with either IFT88 siRNA (AM+siIFT88) or non‐targeting siRNA (AM+siNT) at days 2 and 5 of differentiation induction (both 72 hours post transfection with day 5 receiving a second transfection at day 2). **(B):** Representative images of cilia labeled for acetylated α‐tubulin (green) and IGF‐1Rβ (red) in basal media (BM), adipogenic media (AM), and AM groups with IFT88 siRNA (AM+siIFT88) or non‐targeting siRNA (siNT; AM+siNT). **(C)** Cilia length, **(D)** prevalence, and **(E)** ciliary associated IGF‐1Rβ for each group as described above for day 2 (left) and day 5 (right). *n* = 100–110 cilia per group for (C) and (E), *n* ≥ 5 fields per group with ≥ 10 cells per field for (D). *, *p* < 0.05 versus BM; **, *p* < 0.01 versus BM; ***, *p* < 0.001 versus BM; ##, *p* < 0.01 versus AM; ###, *p* < 0.001 versus AM; +++, *p* < 0.001; Mann–Whitney *U* test. Abbreviations: AM, adipogenic media; BM, basal media; IGF‐1Rβ, insulin‐like growth factor‐1 receptor β; IFT88, intraflagellar transport protein‐88; siNT, non‐targeting siRNA.

**Figure 6:**
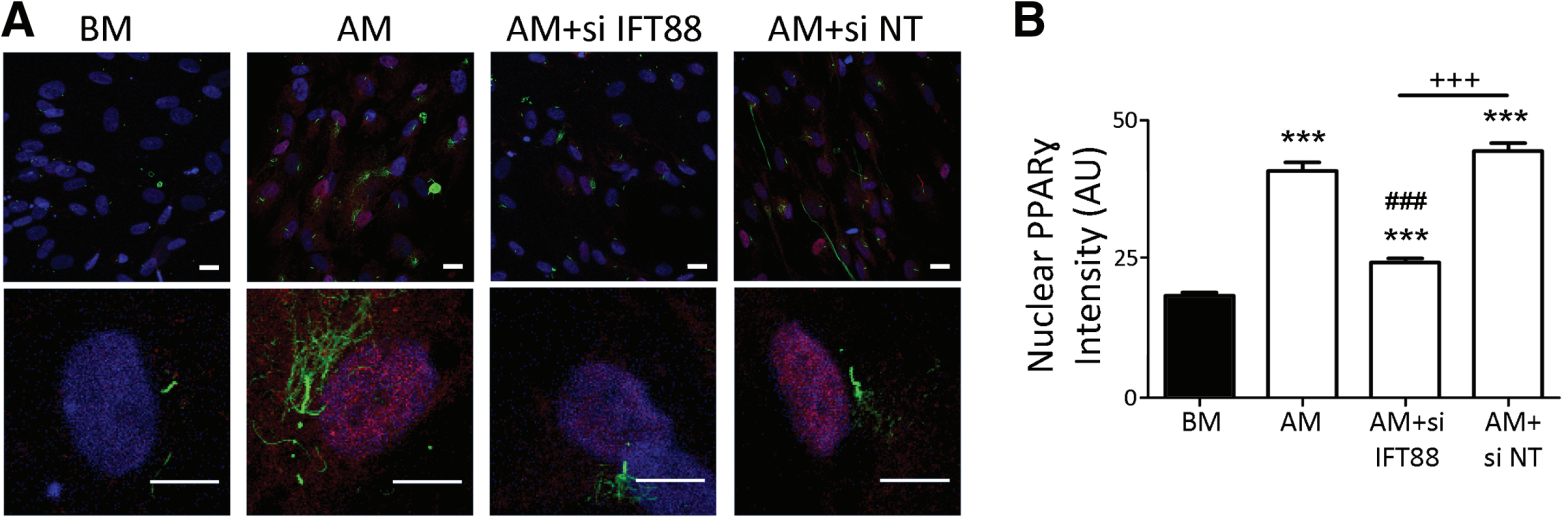
Intraflagellar transport protein‐88 (IFT88) mediated primary cilia elongation is critical for adipogenic differentiation of human mesenchymal stem cells (hMSCs). **(A):** Representative maximum projection confocal images showing hMSCs cultured in basal media (BM), adipogenic media (AM), AM with IFT88 siRNA (AM+siIFT88) and AM with non‐targeting siRNA (AM+siNT). Cells were analyzed at day five of differentiation induction corresponding to 72 hours post second transfection at day 2. Peroxisome proliferator‐activated receptor γ (PPARγ) is shown in red, primary cilia in green, and Hoechst stained nuclei in blue. Scale bar = 20 µm for fields (top) and 10 µm for single cell images (bottom). **(B):** Nuclear PPARγ expression for conditions described above. *n* = 100–110 nuclei per condition. **, *p* < 0.01 versus BM; ***, *p* < 0.001 versus BM; #, *p* < 0.05 versus AM; ###, *p* < 0.001 versus AM; +, *p* < 0.05, +++; *p* < 0.001; Mann–Whitney *U* test. Abbreviations: AM, adipogenic media; BM, basal media; IFT88, intraflagellar transport protein‐88; PPARγ, peroxisome proliferator‐activated receptor γ; siNT, non‐targeting siRNA.

Following our observations that ciliary IGF‐1Rβ localization and nuclear PPARγ levels increase, correlating to primary cilia elongation during adipogenic differentiation, we hypothesized that primary cilia elongation and associated IGF‐1Rβ recruitment to the cilium may be necessary for adipogenic differentiation. To prevent cilia elongation, siRNA targeting *IFT88* was transfected into hMSCs before and during adipogenic differentiation induction (Fig. 5). Low concentrations of siRNA (25nM) were used to prevent cilia elongation without inducing a complete ciliary loss. 24 hours post transfection with IFT88 siRNA and nontargeting control siRNA, hMSC media was replaced with either BM or AM for 2 days before fixation. As nuclear PPARγ levels rose remarkably from day 4 of differentiation (Fig. 2E), some cells were maintained in culture and were subjected to an additional siRNA transfection 72 hours after the first transfection. These cells were then fixed after a further 72 hour correlating to day 5 in BM or AM. Partial silencing of *IFT88* was verified by western blot with a reduction, but not complete elimination of IFT88 protein at both day 2 and day 5 (AM+siIFT88) compared with nontargeting controls (AM+siNT) (Fig. 5A). We successfully prevented cilia elongation in hMSCs cultured in AM with IFT88 siRNA treatment at both days 2 and 5 (Fig. 5B, 5C). There was no difference in cilia prevalence between any groups at day 2, while at day 5 cells treated with IFT88 siRNA (AM+siIFT88) exhibited a reduction of approximately 10% in prevalence compared with those grown in BM (90.1 ± 2.8%) or AM alone (92.7 ± 1.6%) (Fig. 5D). The reduction in cilia length following silencing of IFT88 corresponded with decreased IGF‐1Rβ localization in the primary cilium (Fig. 5B, 5E). Furthermore, hMSCs treated with IFT88 siRNA and cultured in AM exhibited a significant reduction in nuclear PPARγ levels compared with that of AM and nontargeting siRNA (AM+siNT) control groups (Fig. 6A, 6B). This demonstrates that cilia elongation stimulates IGF‐1Rβ ciliary localization and that this is critical for the biochemical induction of adipogenic differentiation in hMSCs.

## Discussion

Primary cilia are involved in mediation of various signaling pathways controlling cell development and metabolism. The requirement for primary cilia in adipogenic and osteogenic differentiation of hMSCs has also been shown by complete knock down of the ciliary trafficking protein IFT88, resulting in loss of primary cilia and decreased expression of adipogenic and osteogenic markers 17. Our data show that primary cilia undergo structural changes during differentiation of hMSCs grown in chemically defined media (Fig. 1). Interestingly, both AM and OM contain dexamethasone and produced an increase in cilia length. Indeed, we have confirmed that this glucocorticoid steroid drives cilia elongation in hMSCs (Fig. 3). By contrast, the presence of TGF‐β3 in CM causes cilia shortening. While we focus on cilia elongation during adipogenic differentiation, changes in cilia structure may also be important for differentiation down various other lineages.

We investigated cilia length and prevalence changes over a 7‐day time course during adipogenic differentiation and found that elongation occurred within the first 2 days (Fig. 2). This elongation was associated with changes in actin stress fiber organization that has previously been linked to cilia elongation via actin tension 35. The time course demonstrates that cilia length was increased in AM throughout the 7 day culture period, although the level of cilia elongation was reduced at days 4 and 7. Nuclear translocation of PPARγ is required for the transcriptional activation of genes upregulated by adipogenic differentiation 36, 37. Nuclear levels of PPARγ were increased at day 2 of adipogenic differentiation but were increased further from day 4 onwards, after maximum cilia elongation had occurred. This suggests that cilia elongation precedes maximal differentiation induction, and is therefore an early event in the differentiation cascade driven directly by the media components.

We next analyzed the effects of AM components on cilia length. AM is composed of dexamethasone, IBMX, and indomethacin with the inclusion of insulin, either from day 0 or after 24 hours 38. We observed that hMSCs displayed the longest cilia in AM without insulin (Fig. 3). In fact, insulin had a negative effect on cilia length when added to AM. Nuclear PPARγ localization increased corresponding to increased cilia length in the absence of insulin but was more enhanced upon supplementation with insulin and in full AM. Insulin alone in BM did not induce cilia elongation and resulted in low nuclear PPARγ levels. Our findings correlate with a previous study that showed accumulation of lipid droplets could occur in hMSCs grown in presence of dexamethasone, IBMX, and indomethacin without insulin, but not in cells grown in media that contained either insulin or IGF‐1 alone 39. Therefore, we propose dexamethasone, IBMX, and indomethacin induce adipogenic differentiation through induction of cilia elongation. This agrees with previous studies that suggest that although insulin is also known to be key mediator of adipogenesis, its effects occur primarily after initiation of differentiation and are instead associated with the maintenance of adipogenic differentiation 33, 34.

IGF‐1 has been shown to be key in adipogenic induction of 3T3‐L1 mouse preadipocytes through binding to its cognate receptor IGF‐1R 34. Furthermore, primary cilia are known to play a role in adipogenic differentiation of mouse preadipocytes through initial activation of cilium localized IGF‐1R upon insulin induction followed by phosphorylation of its downstream effector IR substrate 1 (IRS‐1) and Akt at the basal body 32. Therefore, we hypothesized that cilia elongation is associated with trafficking of IGF‐1R into the cilium. We have shown that there is indeed a significant increase in localization of IGF‐1Rβ in the cilium upon induction of cilia elongation with AM (Fig. 4). Therefore we suggest that adipogenesis is initiated by IGF‐1 activation of IGF‐1Rβ following its recruitment to the cilium associated with cilia elongation. Insulin may then exert a negative feedback on primary cilia length in line with the absence of primary cilia in mature adipocytes 40, 41 and possibly activates pathways for terminal differentiation.

Finally, we demonstrate that preventing primary cilia elongation in hMSCs by subtly inhibiting IFT88 proteins resulted in decreased ciliary IGF‐1Rβ and a subsequent decrease in nuclear PPARγ levels in the presence of AM. Thus, we show for the first time that primary cilia elongation is crucial for adipogenic differentiation of hMSCs through increased IGF‐1R trafficking into the cilium and that blocking this elongation impairs adipogenic induction. The presence of primary cilia are important for regulation of fat metabolism and satiety. Loss of primary cilia or mutations in basal body proteins leading to truncated cilia have previously been shown to lead to obesity 42. Therefore, this study suggests that pharmaceutical manipulation of primary cilia elongation and trafficking could provide a mechanism for preventing adipogenesis and associated obesity.

## Conclusion

In this study, we show that adipogenic differentiation is associated with an increase in cilia length. However, within differentiated cells, there is no correlation between cilia length and ciliary IGF‐1Rβ expression or nuclear PPARγ levels (Supporting Information Fig. S3). This indicates that it is the elongation and associated alteration in cilia trafficking that drives adipogenic differentiation rather than the length *per se*. This is confirmed by disruption of intraflagellar transport with siRNA to IFT88, which prevented induction of cilia elongation blocking IGF‐1Rβ recruitment to the primary cilia and subsequent adipogenesis. These studies provide a new insight into the role of primary cilia in stem cell differentiation and highlight, for the first time, how changes in cilia structure are required for differentiation. This has further implications given the expanding range of physical and biochemical factors, which modulate primary cilia length and may therefore influence adipogenic differentiation of stem cells.

## Author Contributions

M.T.D.: conception and design, collection and assembly of data, data analysis and interpretation, manuscript writing, final approval of manuscript; S.D.T.: conception and design, data analysis and interpretation, manuscript writing, final approval of manuscript; J.T.C.: interpretation of data, final approval of manuscript; J.P.C.: interpretation of data, manuscript writing, final approval of manuscript; M.M.K.: conception and design, financial support, data analysis and interpretation, manuscript writing, final approval of manuscript.

## Disclosure of Potential Conflicts of Interest

The authors indicate no potential conflicts of interest.

## Supporting information

Supplementary Information Figure S1

Supplementary Information Figure S2

Supplementary Information Figure S3

Supplementary Information Figure S4

Supplementary Information Figure S5

Supplementary Information Figure S6
