# Supplementary figures and images for: Adipogenic Differentiation of hMSCs is Mediated by Recruitment of IGF‐1r Onto the Primary Cilium Associated With Cilia Elongation
Source: Stem Cells. 2015 May 21;33(6):1952–61. doi: 10.1002/stem.1975 (PMC4737234; doi:10.1002/stem.1975)

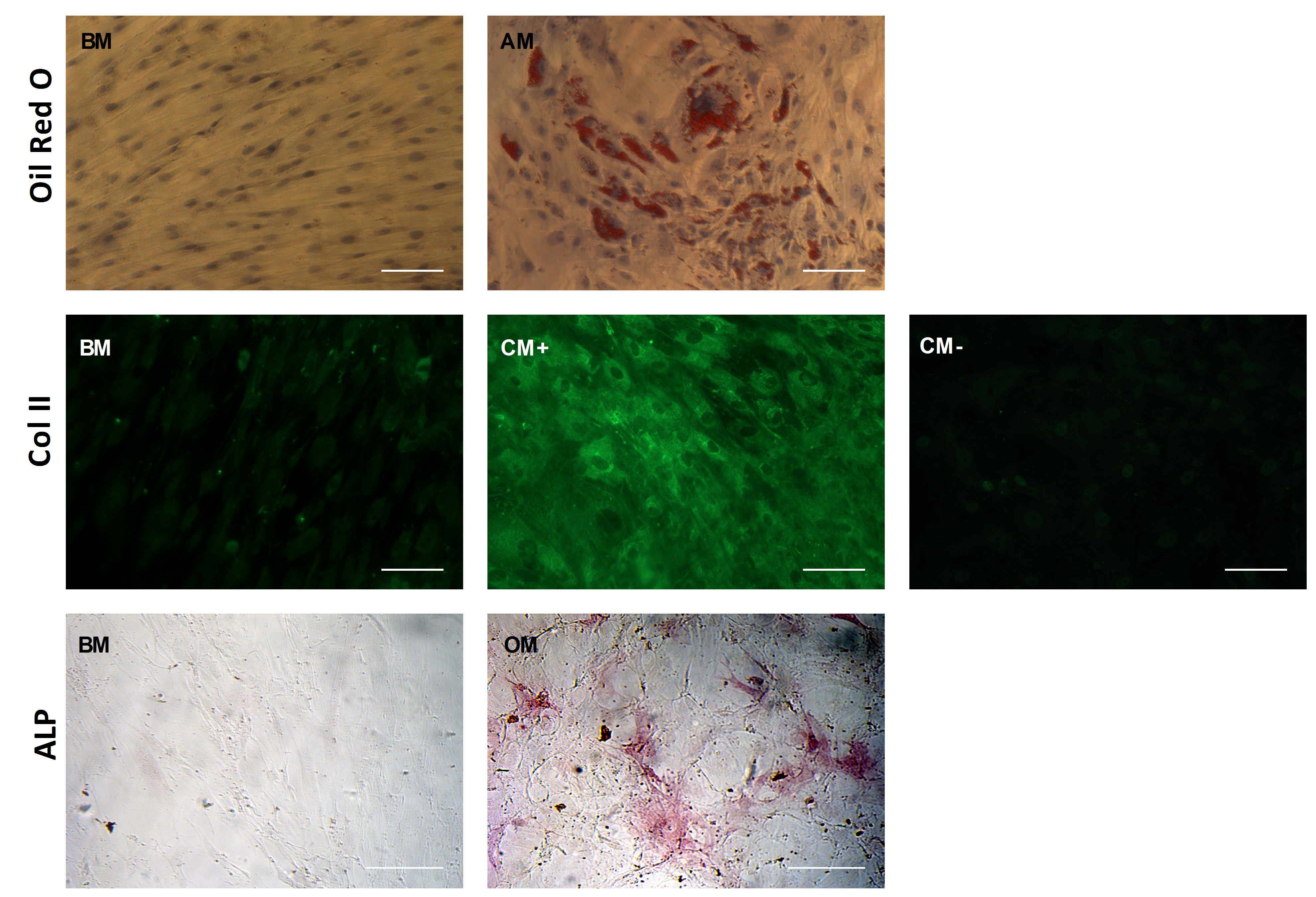

Supplement: Supplementary file 1 — Supplementary Information Figure S1 [file STEM-33-1952-s001.tif]

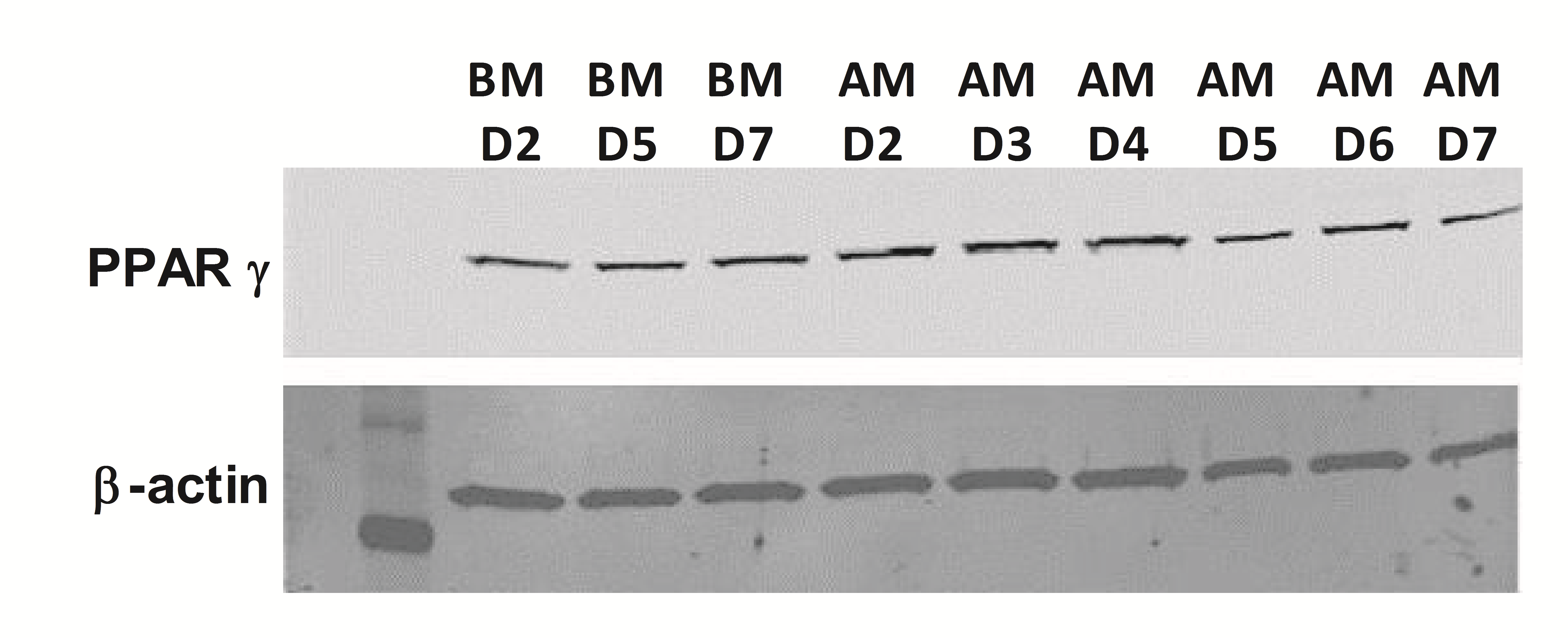

Supplement: Supplementary file 2 — Supplementary Information Figure S2 [file STEM-33-1952-s002.tif]

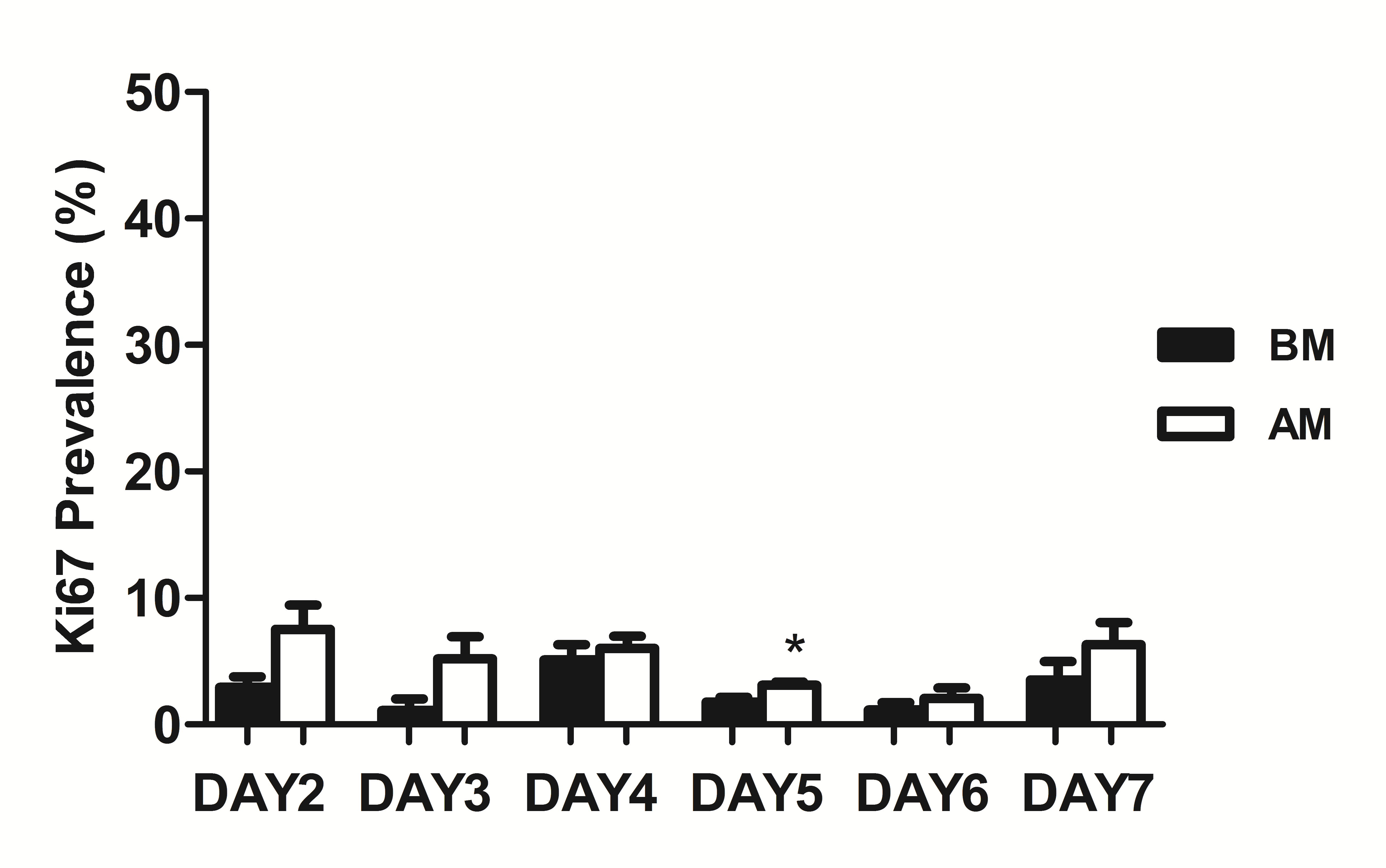

Supplement: Supplementary file 4 — Supplementary Information Figure S4 [file STEM-33-1952-s004.tif]

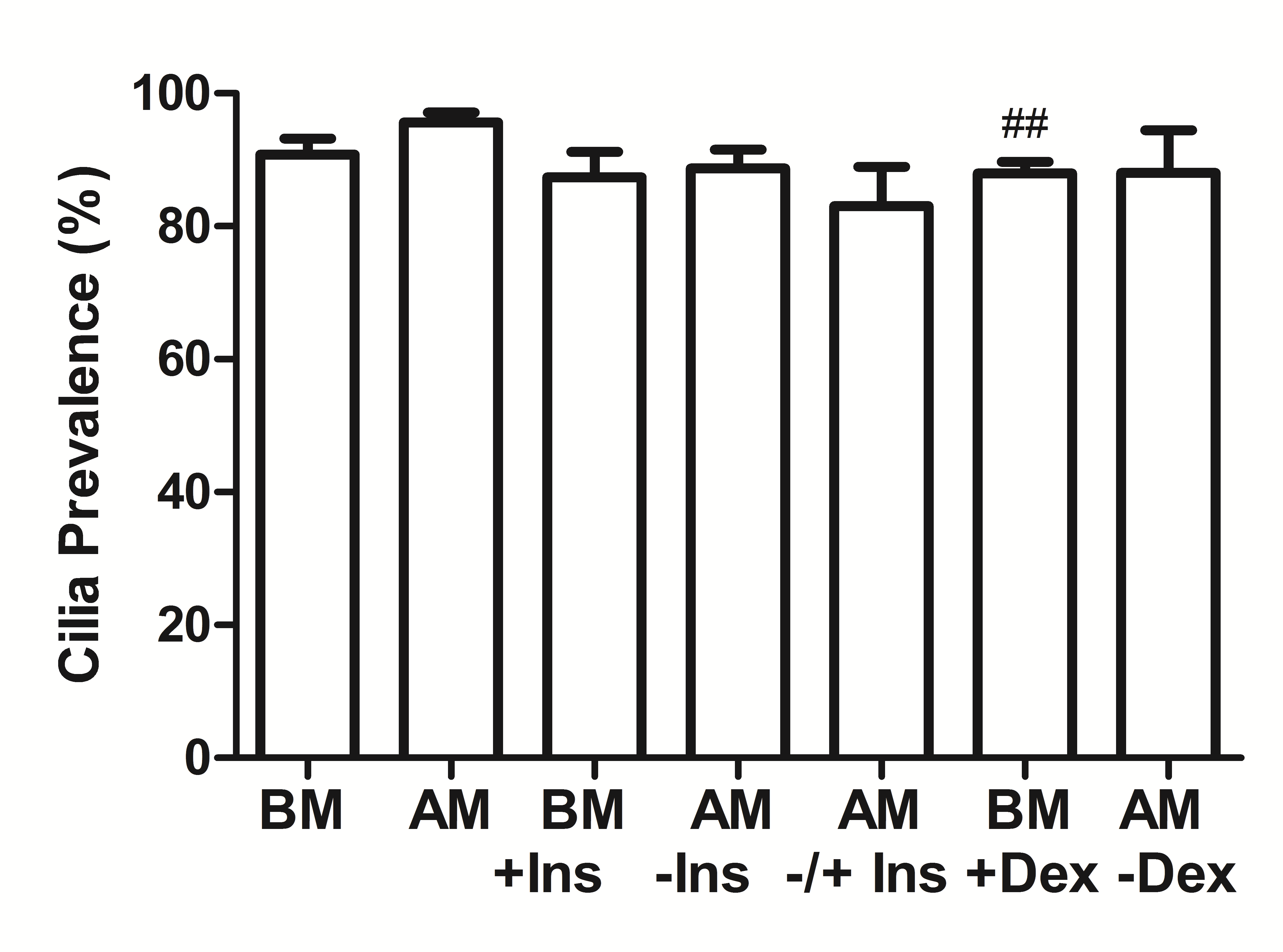

Supplement: Supplementary file 5 — Supplementary Information Figure S5 [file STEM-33-1952-s005.tif]
